# Blood transcriptome resources of chinstrap (*Pygoscelis antarcticus*) and gentoo (*Pygoscelis papua*) penguins from the South Shetland Islands, Antarctica

**DOI:** 10.5808/GI.2019.17.1.e5

**Published:** 2019-03-31

**Authors:** Bo-Mi Kim, Jihye Jeong, Euna Jo, Do-Hwan Ahn, Jeong-Hoon Kim, Jae-Sung Rhee, Hyun Park

**Affiliations:** 1 Unit of Polar Genomics, Korea Polar Research Institute, Incheon 21990, Korea; 2 Department of Polar Life Science, Korea Polar Research Institute, Incheon 21990, Korea; 3 Department of Marine Science, College of Natural Sciences, Incheon National University, Incheon 22012, Korea; 4 Research Institute of Basic Sciences, Incheon National University, Incheon 22012, Korea; 5 Polar Sciences, University of Science & Technology, Daejeon 34113, Korea

**Keywords:** blood, chinstrap, gentoo, immunity, penguin, transcriptome

## Abstract

The chinstrap (*Pygoscelis antarcticus*) and gentoo (*P. papua*) penguins are distributed throughout Antarctica and the sub-Antarctic islands. In this study, high-quality *de novo* assemblies of blood transcriptomes from these penguins were generated using the Illumina MiSeq platform. A total of 22.2 and 21.8 raw reads were obtained from chinstrap and gentoo penguins, respectively. These reads were assembled using the Oases assembly platform and resulted in 26,036 and 21,854 contigs with N50 values of 929 and 933 base pairs, respectively. Functional gene annotations through pathway analyses of the Gene Ontology, EuKaryotic Orthologous Groups, and Kyoto Encyclopedia of Genes and Genomes (KEGG) databases were performed for each blood transcriptome, resulting in a similar compositional order between the two transcriptomes. Ortholog comparisons with previously published transcriptomes from the Adélie (*P. adeliae*) and emperor (*Aptenodytes forsteri*) penguins revealed that a high proportion of the four penguins’ transcriptomes had significant sequence homology. Because blood and tissues of penguins have been used to monitor pollution in Antarctica, immune parameters in blood could be important indicators for understanding the health status of penguins and other Antarctic animals. In the blood transcriptomes, KEGG analyses detected many essential genes involved in the major innate immunity pathways, which are key metabolic pathways for maintaining homeostasis against exogenous infections or toxins. Blood transcriptome studies such as this may be useful for checking the immune and health status of penguins without sacrifice.

## Introduction

Penguins are a monophyletic group within the Spheniscidae family [1]. Approximately 18 species breed sympatrically in several regions within Antarctica and the sub-Antarctic islands [2]. Pygoscelids include three species of penguin (Adélie *Pygoscelis adeliae*, chinstrap *P. antarctica*, and gentoo *P. papua*), and represent congeneric penguins based on their co-occurrence, coexistence, and similarities in their breeding and foraging ecologies [3-5]. Pygoscelids are densely distributed throughout the South Shetland and South Orkney Islands between the latitudes 54°S and 65°S. The chinstraps have a more southern distribution and are located almost exclusively around the Antarctic Peninsula [1,6]. The gentoo have the most northern distribution and forage deeper in the water column. The Adélie and chinstrap are relatively ice-tolerant and depend on ice more than the gentoo [1,6]. In 2017, pygoscelids were placed on the International Union for Conservation of Nature Red List of Threatened Species at the level of Least Concern (http://www.iucnredlist.org). Because their habitats are strongly correlated with Antarctic environmental conditions, fluctuations in the marine environment due to global warming could be the most obvious factor influencing their declining populations [7]. Therefore, consistent monitoring and conservation of pygoscelids is highly recommended.

Penguins serve as important models for studying ecology, the environment, physiology, behavior, reproduction, and population dynamics [8,9]. Antarctica is continuously threated by anthropogenic activities affecting the atmosphere and water including climate change, over-fishing, disease, and tourism [10-12]. Penguins are promising sentinels for monitoring marine pollution and disease in Antarctica given their high trophic position, long lifespan, philopatry, and conspicuous nature [8,9]. While previous studies have analyzed pathogen diversity or toxicant accumulation from penguin blood samples [13-18], to date there have been no published gene expression studies from the blood of any penguin species.

In this study, we analyzed the blood transcriptome of chinstrap (**P. antarcticus**) and gentoo (*P. papua*) penguins using Illumina MiSeq sequencing and a series of bioinformatics tools. Given that blood is highly sensitive to diverse endogenous and exogenous stimulations, including parasites, pathogens, and environmental pollutants, transcriptomic profiling and the measurement of target gene/pathway-specific expression is useful for predicting homeostasis, disease, immunity, and population health status in penguins. Although genomic analyses of penguin blood have been limited, several analytical applications have been conducted on their blood tissues. For example, blood-borne parasitic infections are a potential factor for the mortality of wild penguins [19-21]. Chemical analyses (i.e., dioxins, persistent organic pollutants, polychlorinated biphenyls, and pesticides) of blood samples from penguins have been used as a promising indicator of Antarctic environmental contamination [22-25]. In fact, among birds, blood transcriptome profiling has been successfully employed to predict the homeostasis of immune systems and to analyze transcriptional changes after pathogen or pollutant challenges [26-30]. Thus, genomic information from penguin blood samples will allow the evaluation of their health status using gene expression analyses to determine their immune status.

## Methods

### Ethics statement

All animal handling and experimental procedures were approved by the Animal Welfare Ethical Committee and the Animal Experimental Ethics Committee of the Korea Polar Research Institute (KOPRI).

### Sample collection and Illumina sequencing

Blood specimens (approximately 1 mL) were collected from one individual of each penguin species on King George Island, South Shetland Islands, Antarctica (Table 1). Both species were identified in reference to morphological characteristics and their mitochondrial cytochrome oxidase subunit 1 sequences. Blood samples were immediately transferred to a vial containing RNAlater (Qiagen, Valencia, CA, USA) and stored at –20°C until RNA extraction. Total RNA was extracted using an RNeasy Micro Kit (Qiagen) according to the manufacturer’s instructions and stored in RNAstable (Biometrica, San Diego, CA, USA). The quality and quantity of the total RNA was assessed using the Bioanalyer 2100 (Agilent Technologies, Santa Clara, CA, USA). High-quality mRNA (2 µg) was used to generate a double-stranded cDNA library using poly A selection. Entire experimental reagents and equipments were purchased from Illumina Inc. (San Diego, CA, USA). The NuGEN Encore Complete RNA-Seq Library System (NuGEN, San Carlos, CA, USA) was used to construct paired-end libraries (PE500) of sheared cDNA (500 bp fragments) that were sequenced on an Illumina MiSeq System platform (300 × 2 paired-end reads). Index and adaptor sequences were trimmed using Trimmomatic [31] and low-quality reads were removed using the FASTX toolkit [32] with parameters set to-t = 20,-l = 70, and-Q = 33.

### *De novo* assembly and transcriptome annotation

Low-quality reads (average quality score < 10), adapters, linkers, and polymerase chain reaction primers were removed through quality filtering. High-quality reads were assembled *de novo* using Oases (ver. 0.2.08) with default parameters [33]. The TransDecoder was used to identify coding regions. Unclustered transcripts and the longest sequences were considered unigenes. To identify functional transcripts, all unigenes were searched against the National Center for Biotechnology Information (NCBI) nonredundant database using BLASTx with an E-value threshold of 1.00E^-04^. Gene Ontology (GO) and Kyoto Encyclopedia of Genes and Genomes (KEGG) pathway analyses of all contigs were performed using the Blast2GO sequence annotation tool (ver. 4.0) [34]. BLAST searches and functional domain annotations were conducted using InterProScan within the Blast2GO software package.

Finally, the assembled data were arranged in terms of read length, gene annotation, GenBank number, E-value, species, and species accession number. The mRNA expression levels were calculated using the reads per kilobase of the transcriptome per million mapped reads (RPKM) method [35].

### Data deposition

Raw RNA-seq data were deposited into the NCBI Sequence Read Archive (accession numbers, SRR7874621 for *P. antarcticus* and SRR7874380 for *P. papua*) under the bioproject numbers, PRJNA491756 for *P. antarcticus* and PRJNA491755 for *P. papua*, respectively. The Transcriptome Shotgun Assembly project was deposited into the DDBJ/ENA/GenBank database under accession numbers, GGXL00000000 for *P. antarcticus* and GGXK00000000 for *P. papua*.

## Results and Discussion

Approximately 22.2 and 21.8 million reads were obtained by Illumina sequencing for *P. antarcticus* (accumulated base pairs, 6,675,343,822 bp) and *P. papua* (accumulated base pairs, 6,574,802,598 bp), respectively. In total, 26,036 contigs with an average length of 863 bp and an N50 length of 929 bp were obtained in the *P. antarcticus* blood transcriptome, whereas in the *P. papua* blood transcriptome, 21,854 contigs with an average length of 857 bp and an N50 length of 933 bp were obtained.

**Fig. 1.**
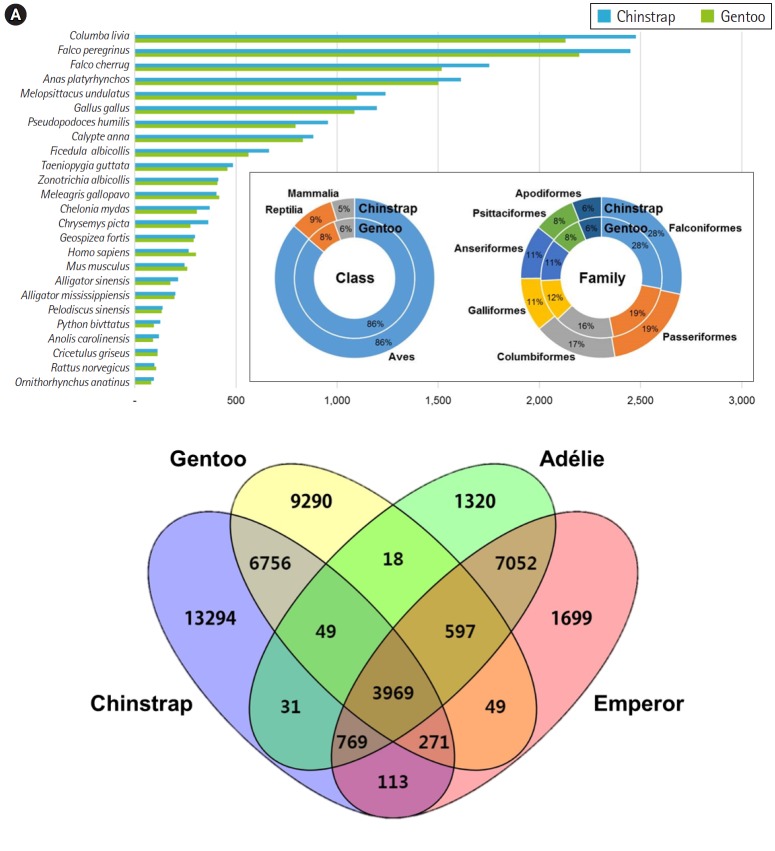
Major BLAST hits matching the chinstrap and gentoo blood transcripts at the phylum and species levels. Each number indicates the quantity of orthologous gene families shared by the indicated genomic database (A). Four-way Venn diagram of orthologous gene conservation in four species of penguins: Adélie (*Pygoscelis adeliae*), chinstrap (*P. antarcticus*), emperor (*Aptenodytes forsteri*), and gentoo (*P. papua*) (B). The diagram was constructed with orthologous genes identified by the best reciprocal hit method with tBLASTx (E-value < 10^–10^) pair-wise ortholog matches. Numbers represent unique or shared genes between penguin species.

Overall, the principal BLAST hits of the penguin transcripts exhibited high similarity to avian genomic information at both class and family levels (Fig. 1A). Of the top hits, 86% of *P. antarcticus* and *P. papua* contigs were homologous to transcripts from the class Aves. At the family level, both contigs showed high similarity to Falconiformes (28%), followed by Passeriformes (19%). These results suggest that the blood sample preparation and Illumina sequencing were successful, as the raw read assembly was undoubtedly characterized as avian. Of the *P. antarcticus* contigs, 2,476 and 2,450 exhibited sequence similarity to transcripts from the rock pigeon *Columba livia* and the peregrine falcon *Falco peregrinus*, respectively. In the case of the *P. papua* contigs, approximately 2,130 and 2,197 showed sequence similarity to transcripts from *C. livia* and *F. peregrinus*, respectively. A previous study generated a total of 15,270 and 16,070 protein-coding genes annotated from Adélie (*P. adeliae*) and emperor (*Aptenodytes forsteri*) penguin genomes [36]. However, the contigs from the current study did not match their genomic information, because only raw sequencing reads of Adélie and emperor penguins were registered in NCBI and annotation data were deposited. Thus, we analyzed orthologous similarity of the penguin transcripts after retrieving the annotated genes of Adélie and emperor penguins [36].

Ortholog comparisons showed extensive similarity within penguins (Fig. 1B). Most transcripts of each penguin matched at least one of the other species. Of the contigs, 3,969 were shared among the four penguins, whereas 1,320, 13,294, 1,699, and 9,290 contigs remained unique to the species Adélie, chinstrap, emperor, and gentoo, respectively. Within the genus *Pygoscelis*, 4,018 contigs were shared among the three penguins within that genus. Overall, 11,045 homologues were observed between the chinstrap and gentoo penguins and 12,387 contigs were identified as homologues between the Adélie and emperor penguins. Although a relatively small number of genes were annotated from the genomes of the Adélie and emperor penguins, this result suggests that blood transcriptomes share many genes encoding for proteins with blood-specific functions.

**Fig. 2.**
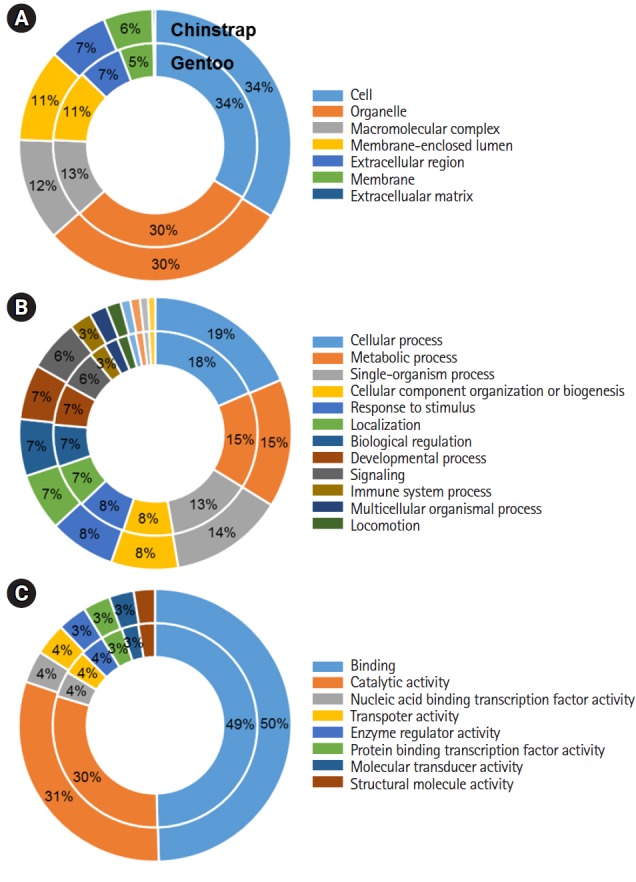
Gene Ontology analyses: cellular components (A), biological processes (B), and molecular functions (C) enriched in the chinstrap and gentoo blood transcriptomes.

The specific GO composition of each principal category (i.e., cellular components, biological processes, and molecular functions) is presented for the *P. antarcticus* and *P. papua* contigs at a Level 2 percentage using default parameters. Overall, similar compositions of GO terms in each category were observed among the blood transcriptomes (Fig. 2). The vast majority of transcripts in the cellular components category were assigned to cells (34%), organelles (30%), and macromolecular complexes (12%–13%) (Fig. 2A). In terms of the biological processes category, many genes were classified as cellular (18%–19%), metabolic (15%), and single-organism processes (13%–14%) (Fig. 2B). Of the GO terms related to molecular function, many genes were categorized as binding (49%–50%) and catalytic activity (30%–31%) components (Fig. 2C). Because blood is one of the most dynamic tissues and encompasses a wide range of cellular metabolisms and numerous exogenous and endogenous factors, the diverse GO assignments analyzed by Blast2GO suggest that penguin blood performs many complex biological functions.

**Fig. 3.**
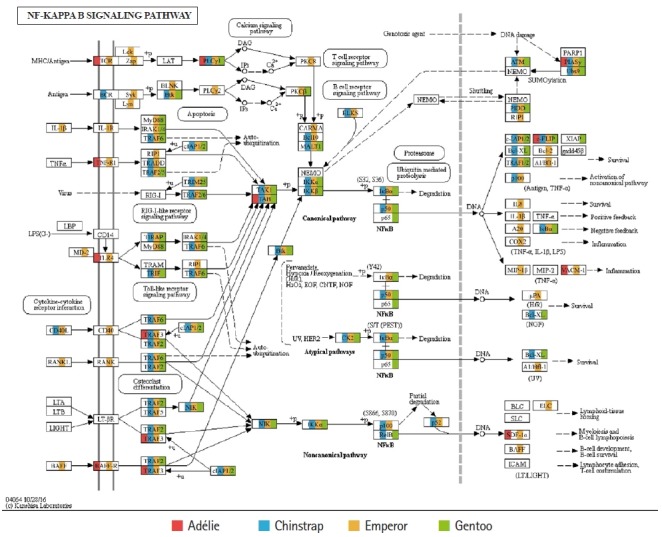
Transcriptional coverage of penguin transcripts within the vertebrate nuclear factor kappa B (NF-κB) signaling pathway (#04064). Matched homologues of each penguin are highlighted in different colors.

**Fig. 4.**
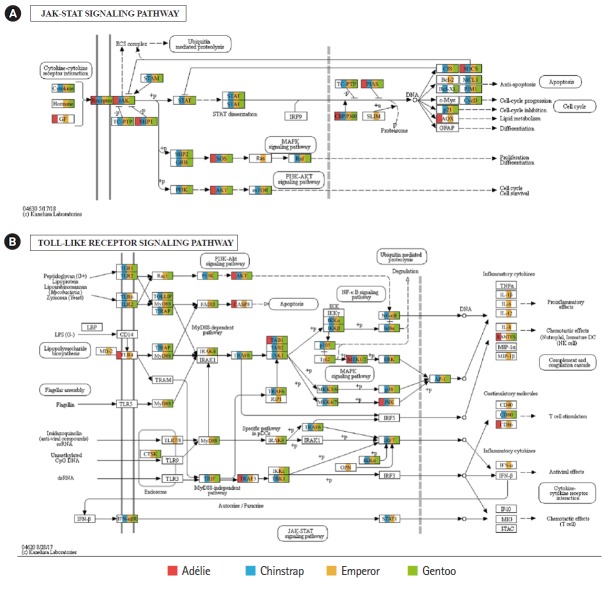
Transcriptional coverage of penguin transcripts on the vertebrate Janus kinase/signal transducers and activators of transcription (JAK-STAT) (#04630) (A) and vertebrate Toll-like receptor (TLR) signaling pathway (#04620) (B). Matched homologues of each penguin are highlighted in different colors.

Of the 20 highest RPKM values, several mRNAs coding for hemoglobin genes were detected in both the *P. antarcticus* and *P. papua* transcriptomes. Because diving birds have developed elevated hemoglobin levels to hold their breath for long periods of time [37], high RPKM values for hemoglobin genes would relate to the capacity for oxygen storage and transfer during routine diving by penguins. Although only limited information is available on the correlation between abundance of hemoglobin gene and oxygen level in penguins, it was identified that hemoglobin concentration is strongly associated with free-diving physiology such as gas exchanges (e.g., oxygen storage, saturation, and depletion) in seals [38,39]. Several major vertebrate canonical signaling pathways such as the nuclear factor kappa B (NF-κB, #04064), Janus kinase/signal transducers and activators of transcription (JAK-STAT, #04630), and Toll-like receptor (TLR, #04620) are important for responding to exogenous pathogens and maintaining homeostasis [40]. In the *P. antarcticus* and *P. papua* blood transcriptomes, the most crucial proteins of innate immunity signaling were detected in the NF-κB (Fig. 3), JAK-STAT (Fig. 4A), and TLR (Fig. 4B) pathways in KEGG pathway analyses. In addition, the transcriptional involvement of immunity-related genes of the TLR, immune deficiency (Imd, #04624), transforming growth factor β (#04350), and interleukin 17 (#04657) signaling pathways were also identified. Overall, the emperor penguin had the greatest number of matched transcripts, whereas the Adélie only had a few proteins from each pathway. A relatively low amount of immunity-related genes were identified in the in the chinstrap and gentoo transcriptomes compared to the emperor penguin. In addition, there was a complete lack of several components from each pathway. This could be explained by the fact that both penguins considered in this study may not be affected by environmental factors or infections. However, the core proteins matched from each pathway suggest that the major innate immune systems are likely expressed and serve as a host defense modulator against exogenous pathological changes in penguin blood. Because the profiling of blood transcriptomes can reflect pathological changes such as immune cell circulation throughout the animal body [41-44] and penguins are sentinel species in the circumpolar region of the Southern Ocean, analyses of blood transcriptomes and hematological parameters could be used as a marker of health status, disease, and response to exposure in penguins.

Here, we presented the first whole-blood transcriptome of two penguin species, the chinstrap and gentoo, and a comparison of pathways involved in the major innate immune systems of four penguin species. Transcriptomic data were successfully obtained from the blood of the penguins and the transcriptomes covered the essential gene repertoire. These data will be useful for understanding the molecular adaptations and immune homeostasis of Antarctic penguins, thereby allowing researchers to assess their health status and resistance to diseases.

## Figures and Tables

**Table 1.** Characteristics of the blood transcriptomes of the chinstrap (*Pygoscelis antarcticus*) and gentoo (*Pygoscelis papua*) penguins in compliance with MIxS standards

Item	*Pygoscelis antarcticus*	*Pygoscelis papua*
Investigation type	Eukaryote transcriptome	Eukaryote transcriptome
Classification	Eukaryota; Opisthokonta; Metazoa; Eumetazoa; Bilateria; Deuterostomia; Chordata; Craniata; Vertebrata; Gnathostomata; Teleostomi; Euteleostomi; Sarcopterygii; Dipnotetrapodomorpha; Tetrapoda; Amniota; Sauropsida; Sauria; Archelosauria; Archosauria; Dinosauria; Saurischia; Theropoda; Coelurosauria; Aves; Neognathae; Sphenisciformes; Spheniscidae; Pygoscelis	Eukaryota; Opisthokonta; Metazoa; Eumetazoa; Bilateria; Deuterostomia; Chordata; Craniata; Vertebrata; Gnathostomata; Teleostomi; Euteleostomi; Sarcopterygii; Dipnotetrapodomorpha; Tetrapoda; Amniota; Sauropsida; Sauria; Archelosauria; Archosauria; Dinosauria; Saurischia; Theropoda; Coelurosauria; Aves; Neognathae; Sphenisciformes; Spheniscidae; Pygoscelis
Project name	*Pygoscelis antarcticus* blood transcriptome sequencing	*Pygoscelis papua* blood transcriptome sequencing
Geographic location name	King George Island (South Shetland Islands), Antarctica	King George Island (South Shetland Islands), Antarctica
Geographic location	62°14′30.8′′S, 58°44′47.7′′W	62°14′30.8′′S, 58°44′47.7′′W
Collection data	17 Jan 2015	17 Jan 2015
Environment (biome)	OMIT_0002267 (Antarctic region)	OMIT_0002267 (Antarctic region)
Environment (feature)	ENVO_00000098 (island)	ENVO_00000098 (island)
Environment (material)	ENVO_01000428 (rocky shore)	ENVO_01000428 (rocky shore)
Tissue type	Blood	Blood
Sequencing method	Pyrosequencing	Pyrosequencing
Sequencing platform	Illumina Miseq	Illumina Miseq
Assembly program	Oases (ver. 0.2.08)	Oases (ver. 0.2.08)
Assembly method	*De novo* assembly	*De novo* assembly
Finishing strategy	Contig	Contig
Bioproject number	PRJNA491756	PRJNA491755
Data accessibility	SRR7874621	SRR7874380
